# Equine Cyathostominae can develop to infective third-stage larvae on straw bedding

**DOI:** 10.1186/s13071-016-1757-1

**Published:** 2016-08-31

**Authors:** Sandy Love, Faith A. Burden, Eoghan C. McGirr, Louise Gordon, Matthew J. Denwood

**Affiliations:** 1 School of Veterinary Medicine, University of Glasgow, Bearsden Road, Glasgow, G61 1QH, UK; 2 The Donkey Sanctuary, Sidmouth, Devon, EX10 0NU UK; 3 Department of Large Animal Sciences, University of Copenhagen, Grønnegårdsvej 8, 1870 Frederiksberg C, Denmark

**Keywords:** Straw, Equid, Donkey, Strongyle, L3, Infection

## Abstract

**Background:**

Domesticated grazing animals including horses and donkeys are frequently housed using deep litter bedding systems, where it is commonly presumed that there is no risk of infection from the nematodes that are associated with grazing at pasture. We use two different approaches to test whether equids could become infected with cyathostomines from the ingestion of deep litter straw bedding.

**Methods:**

Two herbage plot studies were performed in horticultural incubators set up to simulate three straw bedding scenarios and one grass turf positive control. Faeces were placed on 16 plots, and larval recoveries performed on samples of straw/grass substrate over 2- to 3-week periods. Within each incubator, a thermostat was set to maintain an environmental temperature of approximately 10 °C to 20 °C. To provide further validation, 24 samples of straw bedding were collected over an 8-week period from six barns in which a large number of donkeys were housed in a deep litter straw bedding system. These samples were collected from the superficial bedding at 16 sites along a “W” route through each barn.

**Results:**

No infective larvae were recovered from any of the plots containing dry straw. However, infective cyathostomine larvae were first detected on day 8 from plots containing moist straw. In the straw bedding study, cyathostomine larvae were detected in 18 of the 24 samples. Additionally, in the two barns which were sampled serially, the level of larval infectivity generally increased from week to week, except when the straw bedding was removed and replaced.

**Conclusions:**

We have demonstrated that equine cyathostomines can develop to infective larvae on moist straw bedding. It is therefore possible for a horse or donkey bedded in deep litter straw to become infected by ingesting the contaminated straw. This has implications for parasite control in stabled equids and potentially in housed ruminants, and further investigation is required in order to establish the relative infective pressure from pasture versus straw bedding.

**Electronic supplementary material:**

The online version of this article (doi:10.1186/s13071-016-1757-1) contains supplementary material, which is available to authorized users.

## Background

It has been established that the development and/or survival of the infective larval stages of equine strongyles are affected by temperature and humidity in both laboratory experiments [1–3] and field studies [4–13]. These climatic influences on larval development have been extensively reviewed [14]. The presence of equine strongyles on bedding materials of housed horses has been reported [15–17]. In these longitudinal studies, although higher numbers of infective larvae were generally detected during summer months, infective larvae were found on bedding material throughout the 12-month study periods [16, 17]. Our aim was to study the development of strongyle larvae on straw bedding.

The results of three separate, complementary studies are presented in this paper in order to examine and validate different aspects of the laboratory method and findings. A substrate study was used to assess the effect of incubator substrate on larval development, a temperature study was used to assess the affect of temperature within the incubator, and a final bedding study was used to check whether larvae can be recovered from straw in vivo.

## Methods

### Study design

The substrate study was performed in June and July 2014 in West Central Scotland. Separate horse faecal pats were added to three plots within each incubator. The grass/straw in each plot was sampled twice weekly for a 17-day period.

The temperature study was performed in February and March 2015 in West Central Scotland. A donkey faecal pat was added to a single plot within each of three incubators. The straw in each plot was sampled three times per week at regular intervals (Monday, Wednesday, and Friday) over a 22-day period.

The bedding study was performed over a 7-week period from the end of January to early March 2015. Weekly samples were collected in a ‘W’-shape pattern from deep litter straw bedded barns housing a large number of donkeys known to have patent cyathostomine infections. Two of the six available barns were sampled every week for 7 consecutive weeks, two barns were sampled on four occasions, and the remaining two barns were sampled once. The barns were located on properties in the same geographical location in Sidmouth in Devon, England.

### Incubators and equipment

The incubators used in these studies are typically designed for use in horticulture (Bio Green Jumbo Propagators, Chesterfield, England). An electronic thermostat and heating mat controlled the temperature within each incubator, and a high top cover was used to reduce condensation. Each incubator measured 60 × 130 cm and the cover was 50 cm high in the centre. Aluminium trays (dimensions: 50.2 × 119.4 × 2.3 cm) were placed within each incubator to protect the heating mats. Substrate and faecal material was placed onto plastic containers (Van Ness Giant cat Litter Pans,Clifton, NJ, USA), forming plots with dimensions 55.6 × 42.5 × 16.5 cm within the incubator. In the substrate study, the thermostat was set to maintain a temperature of 20 °C within each incubator. In the temperature study, the thermostats were set at 10 °C, 15 °C and 20 °C.

### Individual incubators and plots

For the substrate study, four incubators were used with substrates of clean dry straw, clean watered straw, watered contaminated straw and watered turf. Incubators using contaminated straw contained a bottom layer of deep litter straw bedding collected from a stable of a horse with no patent cyathostomine infection. Each of the four incubators contained three separate plots with the same substrate (12 cm in depth) but using different faecal pats. The faecal pat used for each of the first plots was collected from a horse with no patent cyathostomine infection (to act as a control), the second in each incubator contained faeces from a horse with a low faecal worm egg count (FWEC), and the third plot in each incubator contained faeces from a horse with a moderately high FWEC. Each incubator was set to maintain a constant temperature of 20 °C. Substrates in all incubators (except for the dry straw) were watered daily for the duration of the study.

For the temperature study, each individual plot contained straw and each plot was watered daily throughout the study.

### Watering during incubation

Each individual watered plot was given 100 ml of water per day. The turf plots in the substrate study were given 500 ml of water per plot per day for the first 7 days, and this was gradually reduced to 100 ml per plot per day for the last 5 days of the study. The water was distributed evenly with a watering can.

### Monitoring incubator temperature and humidity

The temperature and relative humidity within each incubator were recorded every 2 h for the duration of the study using the ‘HOBO UX100–011 Temp/RH data logger’ which provides readings within 2.5 % accuracy. One data logger was placed within each incubator. The data were then read from each logger every week using HOBOware version 3.6.2, and the daily averages were calculated from the mean of 12 × 2 hourly temperature recordings.

### Faecal sources

Freshly voided faeces from three adult horses were used to make the faecal pats used for the Substrate Study. One source horse was a 16-year-old gelding sport horse which had not grazed in 7 years and was dosed with ivermectin on three occasions during the first 12 months of being permanently housed. This horse was therefore considered unlikely to be infected with strongyle parasites. The second source horse was a 15-year-old Thoroughbred mare that grazed full-time between June and October during the 4 years prior to this study, and had been dosed with pyrantel pamoate 8 months prior to the study. The third source horse was a 20-year-old Welsh Mountain Pony mare, last grazed 20 months prior to the study and dosed with fenbendazole, then moxidectin, 6 months later.

For 3 weeks prior to the start of the plot study, the FWEC of the source horses were monitored daily. The first (likely uninfected) horse tested negative on all occasions. The second horse had a FWEC that ranged from 75–450 eggs per gram (epg), with a mean of 269 epg. The third horse had a FWEC that ranged from 500 epg to 1350 epg, with a mean of 921 epg. On day 1 of the substrate study the FWEC of the three source horses were 0, 150 and 850 epg, respectively, as determined from the same faecal pats used for the study. A pooled sample of donkey faeces with FWEC of 1,200 epg was used to create the faecal pats for the temperature study.

In the bedding study, weekly samples were collected by walking in a “W” route through each barn picking up pinches of superficial straw from 16 evenly distributed sites. The pinches of straw were collected into separate polythene bags for each barn. Air was manually expressed from the bags, which were then sealed and sent to the University of Glasgow for larval recovery within 48 h of sample collection. Individual faecal samples were also obtained intermittently from the same groups of animals. These samples were individually processed using a modified McMaster method with a minimum sensitivity of 25 epg, and the results were subsequently combined to give group mean animal FWEC.

### Faecal pats

Faecal pats for all plots were made up by mixing 2 kg of faeces from each of the sources (after the addition of 400 ml of water), then making 500 g pats using a 750 ml ‘Lock & Lock’ food container with a 15 cm diameter. Each pat was then placed on top of a piece of mesh with a diameter of 18.5 cm and pore diameter of 2 mm. The pat and mesh were then placed on the incubator substrate (straw or turf).

### Sampling methods and frequency

In the substrate study, each plot was divided into eight equal triangular sections using a wooden frame and string. The frame restricted the sampling area to 38 cm^2^. Each plot was then sampled twice a week for the first 2 weeks, resulting in two sections of each plot being sampled per week. In the third week, the fifth sample was taken as normal, then the remaining three sections in each plot were collected together and pooled to make a single sample (day 17). This resulted in all eight sections of the plots being sampled over 17 days. The sections to be sampled were randomly selected without replacement (i.e. excluding those sections which had already been sampled).

The grass samples were collected by cutting the grass close to the turf, without including the roots. The straw samples were collected by manually lifting up a small handful of the straw and trimming off any excess. The samples were collected across the surface area of the individual triangular section, i.e. from regions both close to and distant from the faecal pat. The grass and straw samples were stored at room temperature in foil dishes and weighed prior to washing for larval recovery. The samples were then re-weighed after being allowed to air dry.

In the temperature study, the straw was sampled three times per week (Monday, Wednesday and Friday) over 22 days, and the sampled sections were randomly selected with replacement, so that some sections were sampled on more than one occasion.

### Larval recovery from herbage/bedding

Samples were put into a washing machine (Mini Washing Machine XPB15–2318, Good Ideas, Tensor Marketing Ltd. Darlington) with 2 l of lukewarm water, and turned through 100 revolutions. The washed material was passed through a coarse mesh sieve and collected in a container, then passed through a 38 μm sieve. Whatever remained in the sieve was then processed using a Baermann technique.

### Faecal culture

At the start of the substrate study, 80 g of faeces from each of the three source horses was put into a separate polystyrene pot, covered with a lid and left at room temperature for 21 days. Any larvae present were recovered using a Baermann technique and identified by microscopy using using the key provided by Soulsby [18].

## Results

No nematodes other than cyathostomine larvae and free-living larvae were identified from the samples in the studies. Free-living larvae were detected in approximately 20 % of the deep litter and turf plots.

### Substrate study

The dry weight of straw samples recovered was between 2.9–7.8 g, and the grass samples weighed from 1.3 to 1.5 g. No infective larvae were recovered from the negative control plots in the substrate study, nor from any plot in the incubator in which the plots contained dry straw (Additional file 1: Figure S1). Larvae were first detected on day 8, and this was from a plot of moist straw at approximately 20 °C and at a relative humidity of 61–70 % (Table 1). Larval development appeared to occur more slowly when the plot was either deep litter straw or turf.

**Table 1:** Summary of results of the substrate study and temperature study

	Substrate study												Temperature study		
Plot herbage	Dry straw			Moist straw			Deep litter straw			Grass turf			Moist straw		
Temperature (°C)	20–21			20–21			20–21			20–21			8–10	15	20–23
Humidity (%)	56–58			61–77			61–76			60–76			70–85	55–65	30–40
FWEC (epg) of faecal source	0	150	850	0	150	850	0	150	850	0	150	850	1,200	1,200	1,200
Total no. of cyathostomine L3/kg recovered	0	0	0	0	977	7,594	0	0	9,996	0	2,500	8,000	0	25,644	21,644
Proportion of viable cyathostomine eggs recovered as larvae	–	0	0	0	0.480	0.182	0	0	0.156	–	0.535	0.021	na	na	na
No. of days to first larval detection	–	–	–	–	8	8	–	–	10	–	17	10	–	13	13

*Abbreviation*: *na* not available

Horse or donkey faecal pats with faecal worm egg counts (FWEC) of 0, 150, 850 or 1,200 eggs per gram (epg) were placed in incubators for approximately 3 weeks and plot herbage samples were collected either twice (substrate study) or three times (temperature study) weekly

From the faecal cultures 0, 1,875 and 38,356 larvae were recovered. The latter two larval recovery rates equated to 3 and 9 % of the total eggs present from 500 g faecal pats with FWEC of 150 epg and 850 epg. The proportion of these potentially viable larvae recovered from the substrate study plots ranged between 0.021–0.535 %.

### Temperature study

The earliest detection of cyathostomine larvae in the temperature study was at day 13, occurring on plots with the lower temperature of 15 °C and humidity of 30–40 % (Table 1; Additional file 1: Figure S1; Additional file 2: Figure S2). Larval culture of the donkey faeces used to create the faecal pats was unsuccessful due to laboratory error, so recovery rates could not be calculated for this study.

### Bedding study

Cyathostomine infective larvae were found on straw bedding samples from each of the six individual donkey barns on at least one occasion. A total of 24 straw bedding samples were collected, 18 of which were positive for cyathostomine larvae and the highest count was 4,551.7 larvae per kg of dry bedding. The range of the group mean faecal egg counts was between approximately 700 and 1,400 epg.

**Fig. 1:**
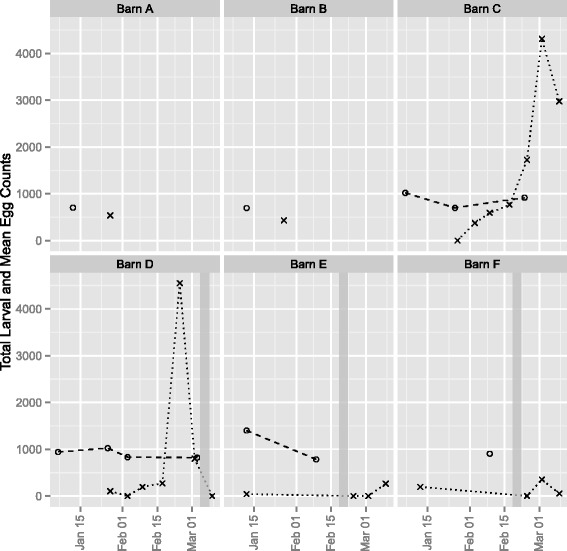
Numbers of cyathostomine infective larvae recovered from weekly straw bedding samples (*crosses*) and mean faecal worm egg count from the corresponding animals (*circles*) from six different barns housing large numbers of donkeys on deep litter bedding (Barn A, *n* = 70 donkeys; Barn B, *n* = 48 donkeys; Barn C, *n* = 140 donkeys; Barn D, *n* = 95 donkeys; Barn E, *n* = 38 donkeys; Barn F, *n* = 43 donkeys). Barn D was cleared out and re-bedded in week 7 immediately after sampling, and Barns E and F were cleared out and re-bedded during week 5 (shown as *grey bars* in the figure)

The mean faecal worm egg counts and corresponding larval recovery from straw bedding during the 8-week period are shown in Fig 1. Barns C and D were sampled on 7 consecutive weeks, during which there was a general increase in straw bedding infectivity. The bedding was cleared out from Barn D after week 7 sampling, and the week 8 sample was negative for cyathostomine larvae. The group mean faecal worm egg counts were relatively consistent and seemed to demonstrate no obvious relationship to the larval counts.

## Discussion

We have demonstrated that equine cyathostominae can develop to infective third-stage larvae on straw bedding both in vitro and in vivo, indicating that the transmission of cyathostomine larvae is possible through the ingestion of bedding. This has clear implications for the health management of housed equine animals, and has potential implications for the management of other grazing species. Similar findings have previously been reported from Czech Republic and Ukraine [15–17].

However, some atypical management practices were employed at the properties where the straw bedding study was performed. On these properties, donkeys were managed in very large groups with a relatively high group mean FWEC. This is in contrast to many equine properties, where only a small proportion of animals have patent cyathostomine infections [19, 20]. The FWEC of the donkeys in the current study were frequently monitored, and targeted anthelmintic dosing was practised using higher FWEC thresholds than would be typically applied on equine properties. We recorded a marked reduction in larval count following the removal of the straw in this study. Following the results of this study, the facility where the bedding study samples were collected adopted the practice of completely renewing the straw bedding approximately every 2 weeks, as a routine preventative measure.

In addition to the findings relating to the management of housed animals, this study has also demonstrated a cheap and effective method of simulating the in vivo development conditions of nematode eggs to infective L3. The incubators generally worked well, creating a range of temperatures and relative humidities. As a result, there is substantial scope for extending these experiments to quantify the mean and variability of parasite development rates under different atmospheric conditions and on varying substrates. Previous studies on cyathostomine development have been performed in a variety of environments on outdoor plots [6, 10, 12, 13]. An advantage of studying parasite larval development under indoor simulated conditions was that the unpredictable effects of prevailing weather conditions (such as rainfall and/or desiccation) could not affect the larval detection and/or recovery. Although the incubators are inexpensive, with a simple design and function, they clearly do not represent a precise simulation of management conditions where factors such as ongoing faecal contamination, mechanical disturbance of bedding and equine urine contamination may affect cyathostomine larval development. One specific limitation of the equipment used in the studies reported here was the inability to reduce the temperature within the incubators to below the ambient temperature. This may pose a potential problem in the use of these methods during relatively warm summer months.

The work presented here is intended as a proof of concept rather than an investigation of cyathostomine biology per se, yet the observations on development rates support the intention that the study conditions were realistic representations of practical management conditions. Furthermore, the findings of the study reported here compared well with previous studies on cyathostomine development with regards to the duration of development to infective third-stage larvae. At a temperature of approximately 20 °C, and at a relative humidity of approximately 60–80 %, infective larvae were present in approximately 1 week. The development of larvae was observed to be slightly slower in donkey faeces, but due to the limited sample size and varying humidity between the substrate and temperature studies, it was not possible to determine if this difference was due to the parasites, atmospheric conditions, or simply chance. It is interesting that there was no apparent difference between the development rate of larvae kept at 15 °C and 20 °C, although it is possible that the reduction in humidity at higher temperatures offset any increase in growth rate due to the higher temperature. It is also interesting that the number of larvae recovered from turf was not qualitatively higher than that recovered from straw, as turf would be expected to present the more natural (and therefore presumably more suitable) habitat for the parasites. The most likely explanation is that the relatively small sample size and high variability between larval counts obscured the difference between the substrates, and that a larger study would indeed show a higher average larval count on turf. However, it is also possible that daily watering resulted in more larvae being washed clean from the grass compared to straw, or that a difference in the densities of the two materials resulted in a larger volume of straw giving the same dry weight as a smaller volume of grass. Further study of these potential factors is therefore required before the larval count method can be considered to produce equivalent results from dissimilar substrates.

From the faecal cultures, it was established that the viability of the strongyle eggs in the substrate study faecal pats was between 3–9 %. Previous plot studies on cyathostomine development reported cyathostomine egg viability of 96 % [10]. There is no clear explanation for the comparatively low viability of cyathostomine eggs in the current study, but recovery rates of the order of 0.3 % observed from straw under the optimal conditions suggest that only approximately 10 % of the potentially viable larvae are capable of developing to L3 within the normal development time on straw.

## Conclusions

We have demonstrated that equine cyathostominae can develop to infective larvae on moist straw bedding. It is therefore possible for a horse or donkey bedded in deep litter straw to become infected by ingesting the contaminated straw. This has implications for parasite control in stabled equids and potentially in housed ruminants, and further investigation is required in order to establish the relative infective pressure from pasture versus straw bedding.

## Additional files

Additional file 1: Figure S1. Numbers of cyathostomine infective larvae recovered from serial samples taken over a 17-day period from four different substrate incubators (deep litter straw, dry straw, watered straw and grass turf). Within each of the four incubators, three plots were set up using faecal sources of 0 epg, 150 epg and 850 epg, respectively. (PDF 5 kb) Additional file 2: Figure S2. Numbers of cyathostomine infective larvae recovered from serial samples taken over a 22-day period from three straw bedding plots incubated at 8–10 °C, 15 °C and 20–23 °C, respectively. (PDF 5 kb)

## Abbreviations

FWEC: Faecal worm egg count
epg: Eggs per gram
