# Supplementary figures and images for: Equine Cyathostominae can develop to infective third-stage larvae on straw bedding
Source: Parasit Vectors. 2016 Aug 31;9(1):478. doi: 10.1186/s13071-016-1757-1 (PMC5006614; doi:10.1186/s13071-016-1757-1)

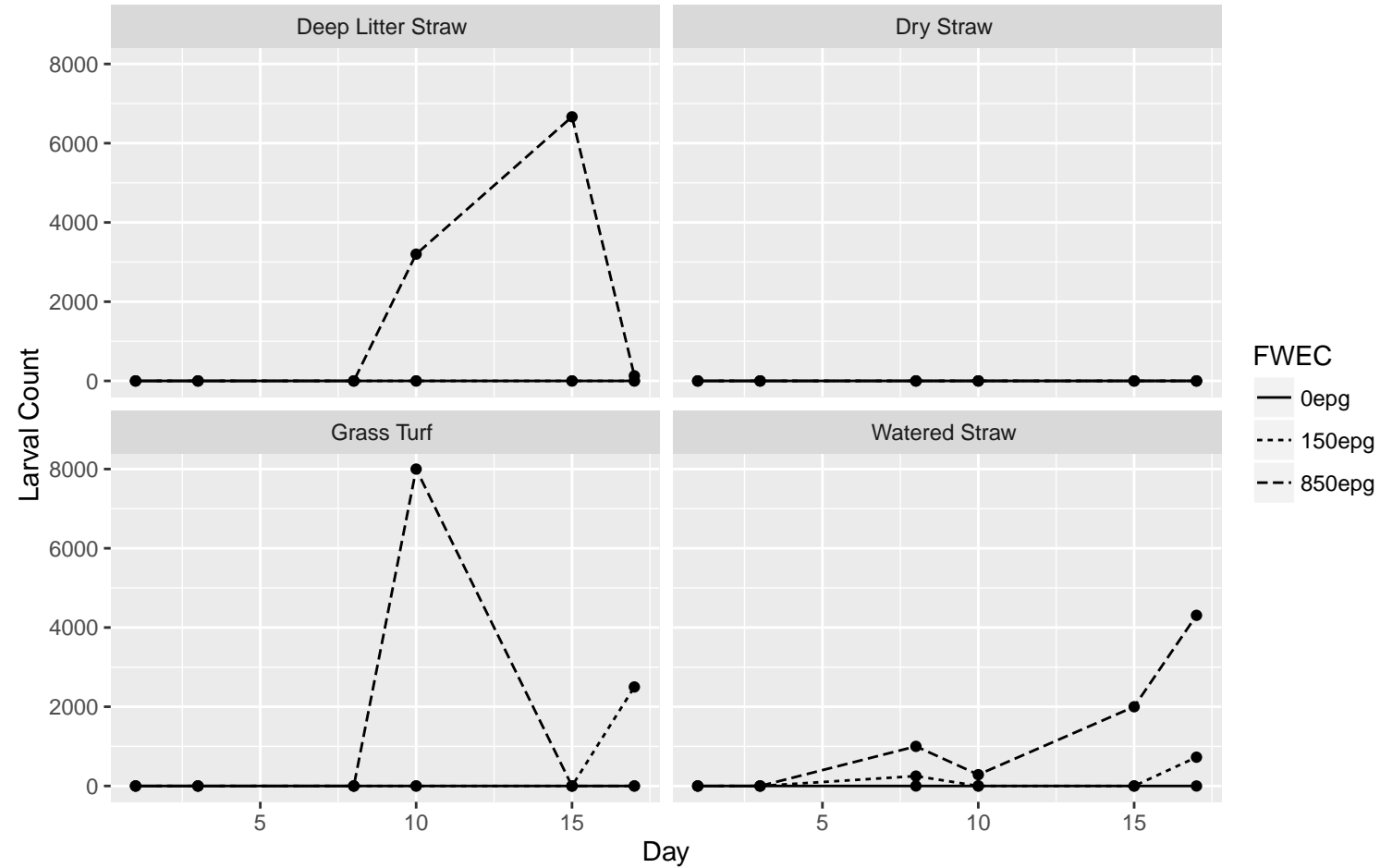

Supplement: Additional file 1: Figure S1. — Numbers of cyathostomine infective larvae recovered from serial samples taken over a 17-day period from four different substrate incubators (deep litter straw, dry straw, watered straw and grass turf). Within each of the four incubators, three plots were set up using faecal sources of 0 epg, 150 epg and 850 epg, respectively. (PDF 5 kb) [file 13071_2016_1757_MOESM1_ESM.pdf]

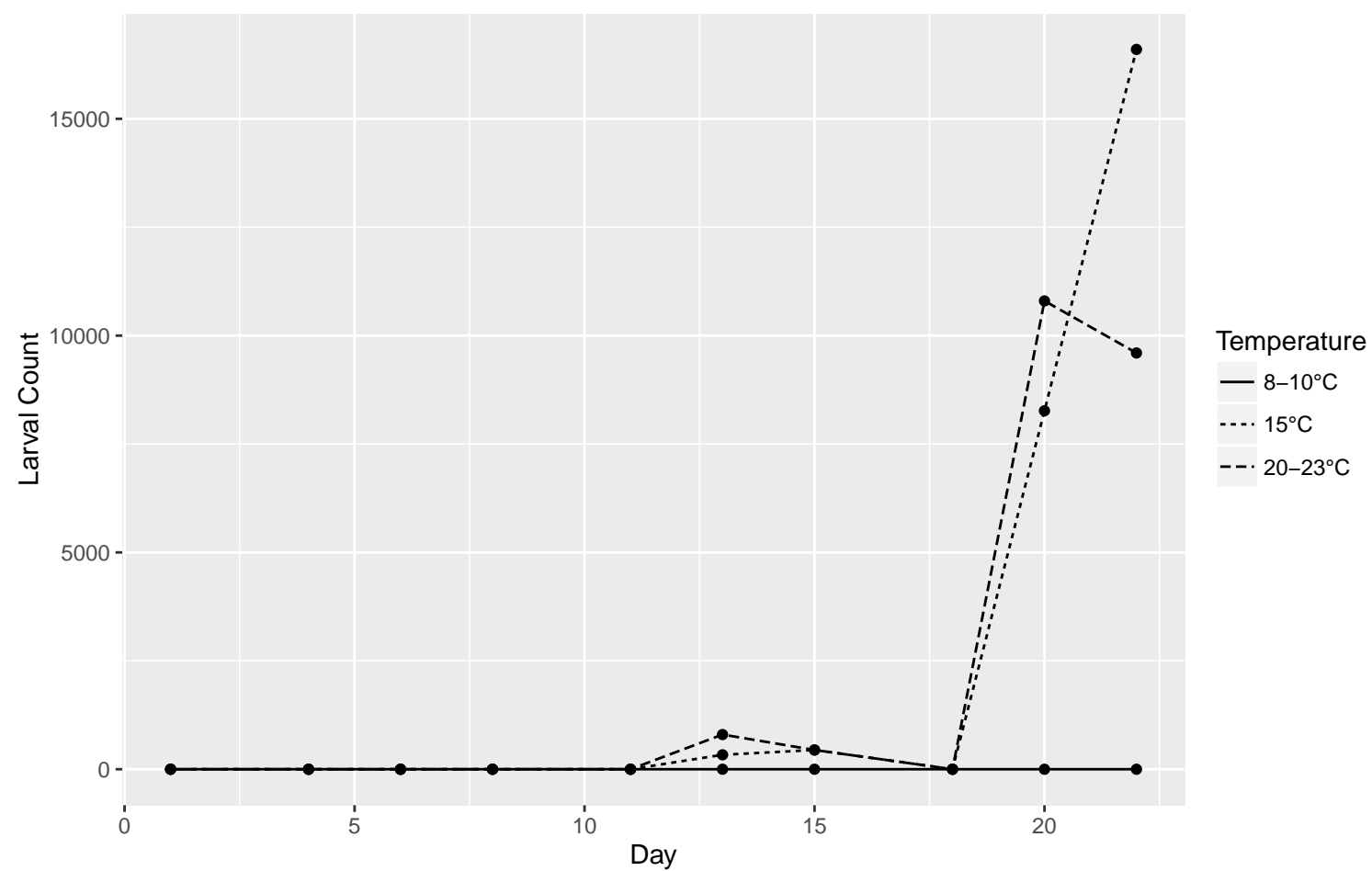

Supplement: Additional file 2: Figure S2. — Numbers of cyathostomine infective larvae recovered from serial samples taken over a 22-day period from three straw bedding plots incubated at 8–10 °C, 15 °C and 20–23 °C, respectively. (PDF 5 kb) [file 13071_2016_1757_MOESM2_ESM.pdf]
